# Percutaneous Kidney Puncture Using a Novel Contrast-enhanced Ultrasound-guided Technique: First Swine Model *In Vivo* Experience

**DOI:** 10.1016/j.euros.2025.09.001

**Published:** 2025-09-22

**Authors:** Donglai Shen, Shoupeng Li, Hairui Chen, Wei Wang, Haomin Liang, Chong Zhang, Weiguo Xie, Jisong Tian, Xu Zhang, Bingding Huang, Haixing Mai

**Affiliations:** a Department of Urology, the Third Medical Center, Chinese PLA General Hospital, Beijing, China; b Department of Ultrasound, the First Medical Center of PLA General Hospital, Beijing, China; c The Fifth Clinical School of Medicine, Anhui Medical University, Hefei, China; d College of Big Data and Internet, Shenzhen Technology University, Shenzhen, China; e Wuerzburg Dynamics Ltd., Shenzhen, China; f The Second Clinical Medical School, Lanzhou University, Lanzhou, China

**Keywords:** Percutaneous nephrolithotomy, Precise puncture technique, Contrast-enhanced ultrasound, Free artificial hydronephrosis, Swine

## Abstract

**Background and objective:**

This study introduces a novel dual contrast-enhanced ultrasound (D-CEUS) technique and evaluates its feasibility and safety for an *in vivo* puncture of the nondilated renal collecting system during percutaneous nephrolithotomy.

**Methods:**

Percutaneous punctures were performed in six swine, with each animal receiving six bilateral renal punctures (upper, middle, and lower calyces). D-CEUS guidance was used for the left kidney, and conventional ultrasound was used for the right kidney. The outcomes included the first-attempt success rate, preparation time, puncture time, correct puncture rate, and vascular injury incidence.

**Key findings and limitations:**

D-CEUS demonstrated statistically significant improvements compared with conventional ultrasound, including a higher first-attempt success rate (94.4% vs 61.1%) and correct puncture rate (100% vs 66.7%), lower vascular injury (0% vs 27.8%), and shorter puncture time (median 26 vs 45 s). However, as this is a preclinical animal study, limitations remain, and clinical validation is needed. All punctures were performed under general anesthesia, followed by tract dilation to 24F along an inserted guidewire.

**Conclusions and clinical implications:**

D-CEUS provides real-time visualization of vascular anatomy and needle trajectory in a nondilated collecting system, enhancing puncture accuracy. Its ability to deliver contrast directly through the puncture needle enables precise stone localization and supports the procedure’s potential to improve the safety and efficiency of percutaneous nephrolithotomy.

**Patient summary:**

In this report, we applied a novel contrast-enhanced ultrasound-guided technique to achieve more precise renal puncture targeting. We found that the application of the novel technique performs better than conventional ultrasound guidance in percutaneous nephrolithotomy.

## Take Home Message

We demonstrated that the dual contrast-enhanced ultrasound technique improves puncture accuracy and precise stone targeting during percutaneous nephrolithotomy by enabling real-time simultaneous visualization of the vasculature, puncture needle, and collecting system in a nondilated system.

## 1 Introduction

Urolithiasis is one of the most common urinary tract diseases and has shown a steady increase in prevalence in recent years [1]. Percutaneous nephrolithotomy (PCNL) remains the gold standard for treating large and complex stones in the upper urinary tract [2]. However, the success of PCNL depends heavily on operator expertise and a thorough three-dimensional anatomical understanding, particularly for an accurate calyceal puncture, which is critical for optimal outcomes [[3], [4], [5]].

Although a fluoroscopic-guided puncture is effective, it exposes patients and staff to ionizing radiation [6]. Ultrasound (US) guidance avoids radiation exposure and is more cost effective [7], but visualization of target calyces in a nondilated collecting system is often suboptimal [8,9]. Artificial hydronephrosis may enhance visibility; however, elevated renal pressure carries additional risks [9]. Contrast-enhanced ultrasound (CEUS) uses gas-filled microbubble agents and avoids ionizing radiation. It offers superior real-time visualization to conventional US [[10], [11], [12]]. CEUS is safe, affordable, and used widely in various clinical settings.

Intracavitary CEUS (IC-CEUS) has been applied to assess physiological tracts, such as vesicoureteral reflux and fallopian tube patency [13,14]. It was first introduced in PCNL to identify obstruction sites [15]. Although off-label, IC-CEUS has shown promise in guiding a puncture in nondilated systems by a retrograde injection of contrast through a ureteral catheter, improving puncture accuracy and stone clearance [16]. Several studies have demonstrated its higher first-attempt success rate and shorter puncture times than conventional US [[15], [16], [17]].

**Fig. 1:**
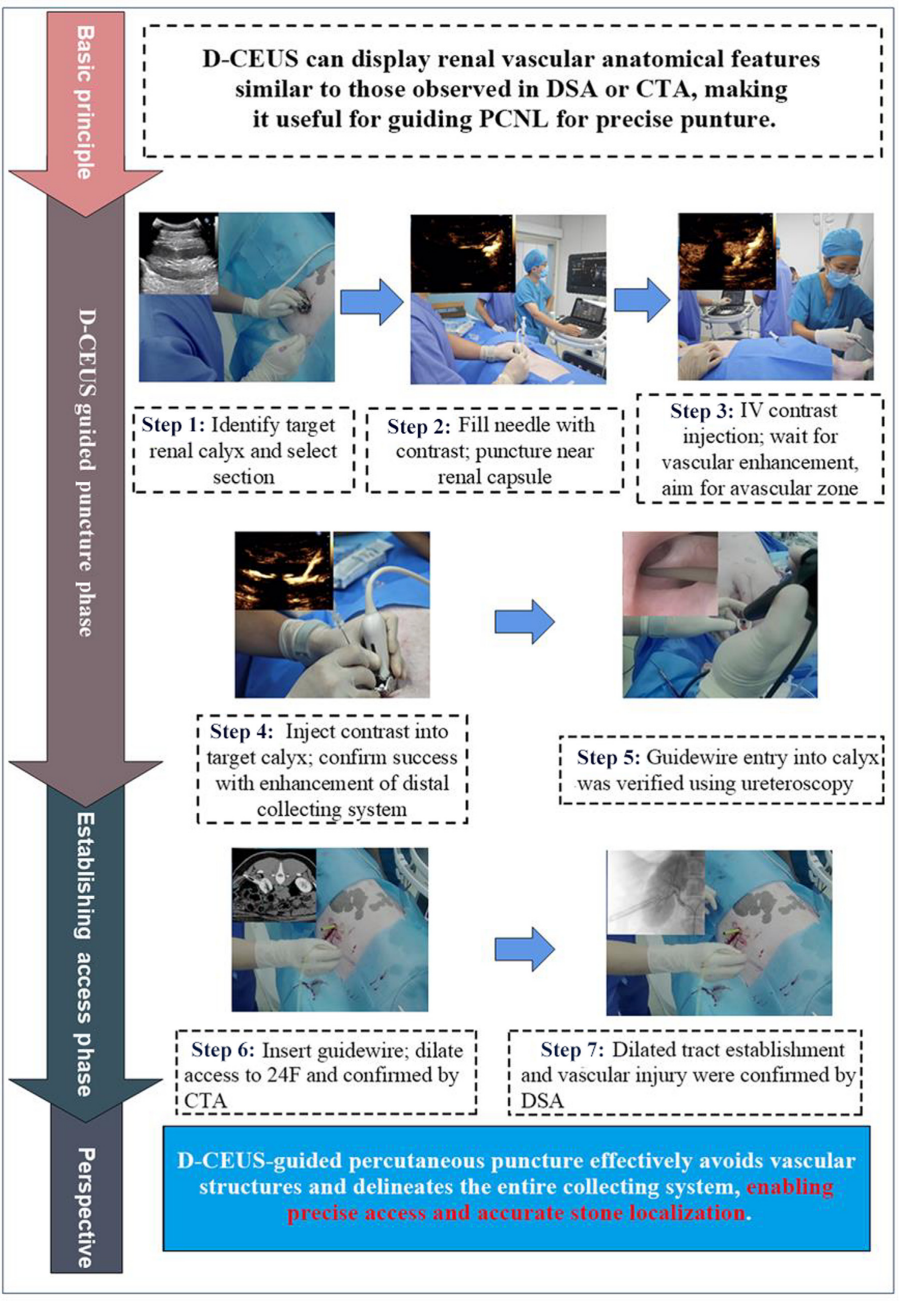
Graphical abstract of a novel guidance technique for PCNL. CTA = computed tomographic angiography; D-CEUS = dual contrast-enhanced ultrasound; DSA = digital subtraction angiography; IV = intravenous; PCNL = percutaneous nephrolithotomy.

While IC-CEUS excels at visualizing tract obstruction, it lacks the capability to image blood vessels, which is critical for avoiding injury to blood vessels during a puncture. To address this issue, we developed a novel dual CEUS (D-CEUS) technique that provides real-time guidance through two sequential phases: First, intravenous CEUS (IV-CEUS) is used for vascular mapping. Then, IC-CEUS is performed via the puncture needle to visualize the collecting system and localize the stone. This dual-modality approach improves trajectory planning and reduces the risk of vascular injury (Fig. 1).

## 2 Materials and methods

### 2.1 Experimental patients

This study involved six healthy female pigs between 4 and 12 mo of age and weighing 55–70 kg. Female animals were chosen due to their comparable renal anatomy and because retrograde ureteroscope placement in males is technically more difficult during PCNL. All pigs were nonpregnant, nonlactating, and free of coagulopathy, major organ dysfunction, anatomical abnormalities along the planned puncture path, or intolerance to the prone position.

The experimental protocol was approved by the Ethics Committee for Experimental Animals of Guangzhou Boshi Medical Technology Co., Ltd, Foshan, China (BSYXIA039). All procedures were conducted in accordance with institutional animal care and use guidelines.

### 2.2 Preoperative preparation procedure

All pigs fasted for 12 h before surgery. Subcutaneous atropine sulfate (0.5 mg/kg) was administered to reduce respiratory secretions. Intravenous thiopental sodium (250 mg) was administered via a marginal ear vein catheter to induce anesthesia. After endotracheal intubation, general anesthesia was maintained with a propofol and dexmedetomidine infusion supplemented with isoflurane for muscle relaxation. Subcutaneous meloxicam (10 mg) was administered for analgesia. Intraoperative monitoring included an electrocardiogram via limb leads and a pulse oximeter on the tongue.

Digital subtraction angiography (DSA) is a well-established and reliable technique for visualizing blood vessels in detail using intravenous contrast agents. In this study, renal DSA was performed via femoral artery catheterization to evaluate the renal vasculature. Prior to surgery, all pigs underwent DSA and computed tomographic angiography (CTA; slice thickness and interval: 1.0 mm; Royal Philips, Amsterdam, The Netherlands) to randomly select the target kidney and calyx, and assist in planning the puncture trajectory.

### 2.3 Description of the surgical procedure

Each pig underwent six bilateral renal punctures (upper, middle, and lower calyces) performed by an experienced surgeon. The left kidney received a D-CEUS–guided puncture (D-CEUS guided), while the right kidney underwent a conventional US–guided puncture (conventional US guided).

#### 2.3.1 D-CEUS–guided group

**Fig. 2:**
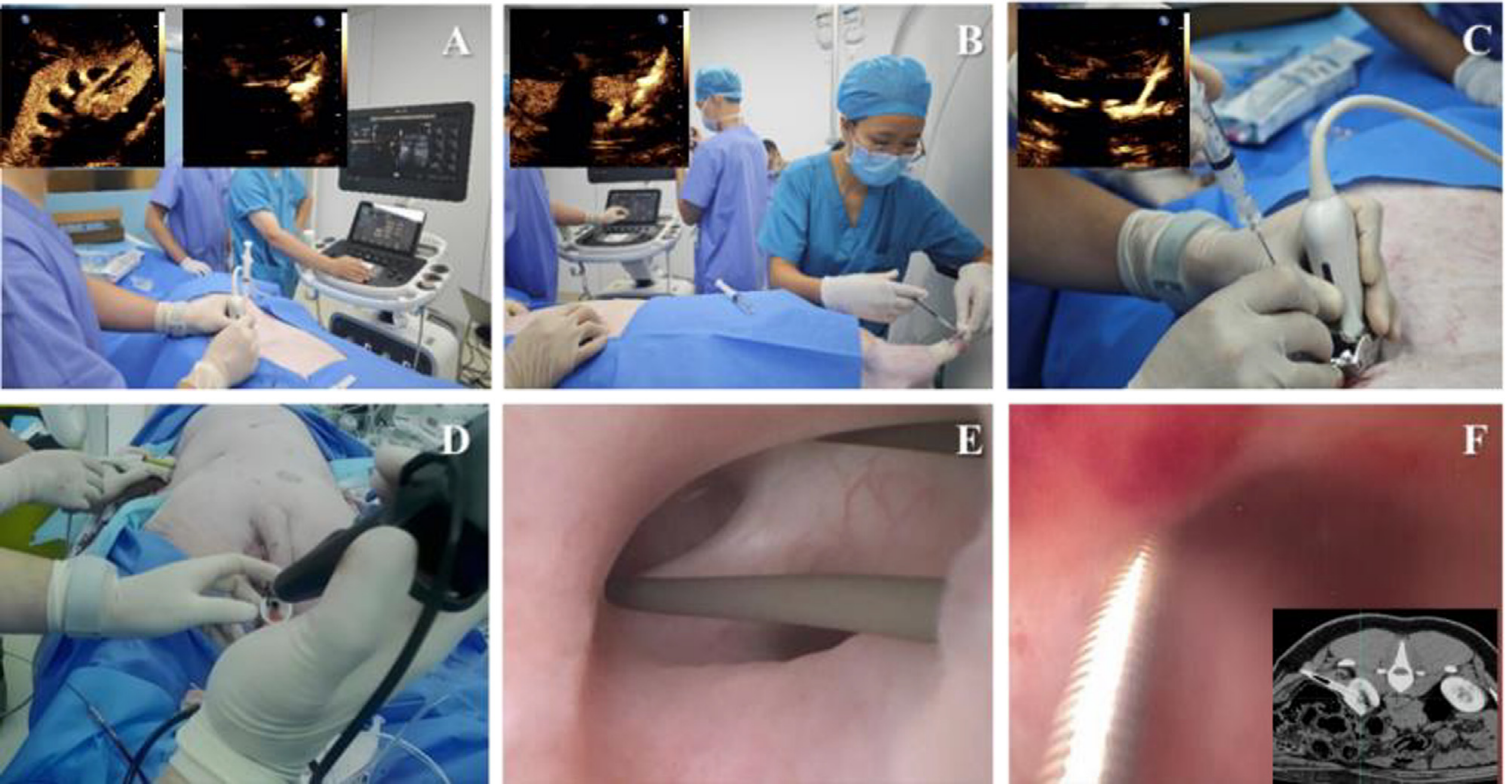
Stepwise procedure for percutaneous access to the renal collecting system in an *in vivo* model. (A) The contrast-filled needle tip was advanced to the renal capsule. (B) Following intravenous injection of 0.5 ml contrast agent, vascular filling was observed and puncture was performed through relatively avascular areas. (C) Upon needle entry into the target calyx, diluted contrast agent (1:100) was injected through the needle to visualize the collecting system. (D and E) Guidewire placement was confirmed via both retrograde and antegrade ureteroscopy. (F) CTA confirmed successful establishment of the dilated tract. CTA = computed tomographic angiography.

Conventional US was first used to locate the kidney and identify the maximal longitudinal axis, and to determine the puncture entry point. In the CEUS mode, an 18 G needle prefilled with diluted contrast agent (SonoVue, at a dilution of 1:100) and connected to a syringe containing 1 ml of the same solution was advanced to the renal capsule (Fig. 2A). Then, 0.5 ml of the standardized contrast agent was administered intravenously. Once the renal vasculature was clearly visualized, the needle was advanced along the bisector of the angle formed by the two arcuate arteries (Fig. 2B).

Once the needle entered the target calyx, the diluted contrast agent was slowly injected through the needle lumen to visualize the collecting system. A successful puncture was indicated by a clear delineation of the target renal calyx and pelvis (Fig. 2C). Then, a guidewire was inserted, and its position was confirmed by flexible ureteroscopy (Fig. 2D and E). Stepwise dilation was performed to establish a 24 F percutaneous nephroscope tract.

#### 2.3.2 US guidance group

Once the maximal longitudinal axis and entry point were identified, an 18 G needle was advanced toward the target calyx under conventional US. A successful puncture was confirmed by fluid efflux from the needle or by directly visualizing the needle tip via flexible ureteroscopy. Tract dilation followed the same steps as in the D-CEUS–guided group.

**Fig. 3:**
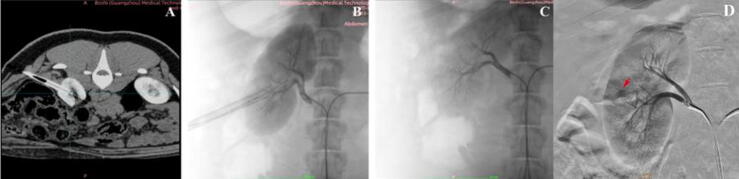
Postoperative CTA and DSA imaging: (A and B) dilated tract establishment confirmed by CTA and DSA, (C) assessment of vascular injury by DSA after tube removal in the D-CEUS–guided group, and (D) assessment of vascular injury in the conventional US-guided group. The red arrow indicates the bleeding site. CTA = computed tomographic angiography; D-CEUS = dual contrast-enhanced ultrasound; DSA = digital subtraction angiography; US = ultrasound.

In both groups, tract establishment was confirmed by CTA and DSA (see Fig. 3A and B). Postoperative vascular injury was assessed using DSA after tube removal with the same protocol as in preoperative imaging (Fig. 3C and D).

### 2.4 Outcome measurement

The following outcomes were compared between the D-CEUS and conventional US groups:

1. Preparation time: the time from the initial identification of the kidney axis to when the puncture needle reaches the renal capsule, ready for insertion.
2. Puncture time: the time from when the needle was ready for a puncture to when the puncture was confirmed as successful.
3. First-attempt success rate: the percentage of successful punctures completed without needle adjustment.
4. Correct puncture rate: the percentage of punctures with a trajectory targeting the fornix or papilla of the posterior calyx that are within 20° posterior to the kidney’s frontal plane [18]; deviations beyond this range were considered incorrect.
5. Vascular injury rate: incidence of vascular injury confirmed by DSA.

### 2.5 Statistical analysis

Continuous variables were presented as median (minimum – maximum), and categorical variables were presented as counts (*n*) and percentage (%). Statistical analyses were performed using SPSS version 22.0 (IBM Corporation, Armonk, NY, USA), including the Mann-Whitney *U* and Fisher exact tests. A *p* value of <0.05 was considered statistically significant.

## 3 Results

**Fig. 4:**
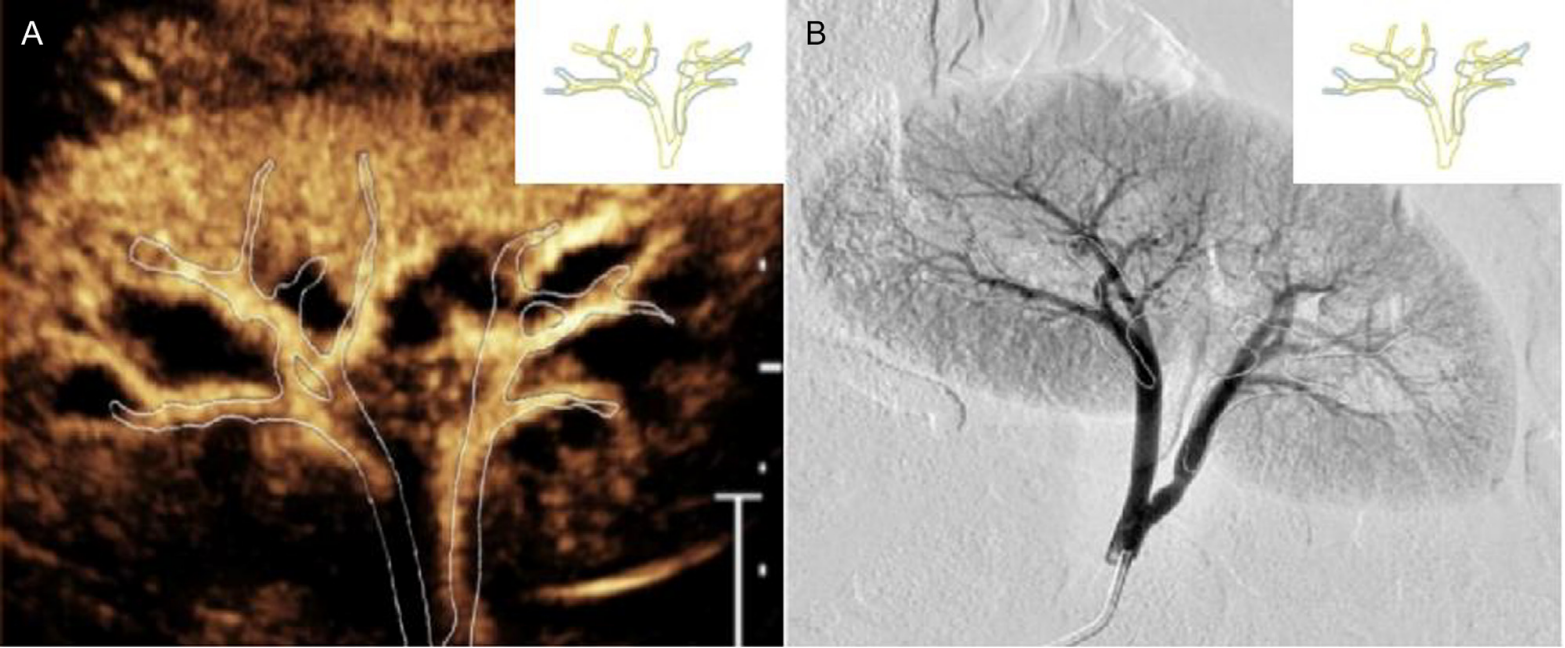
Visualization of renal cortical vasculature in a nondilated collecting system using (A) CEUS and (B) DSA. The vascular anatomy visualized by CEUS closely matched that observed in preoperative DSA, demonstrating consistent alignment of renal vascular structures. CEUS = contrast-enhanced ultrasound; DSA = digital subtraction angiography.

As shown in Fig. 4, CEUS enables real-time visualization of renal vascular structures in a nondilated collecting system, comparable with preoperative DSA, confirming the feasibility of the novel D-CEUS technique.

All 18 punctures in the D-CEUS–guided group were completed successfully without needle adjustment and complications (Table 1 and Fig. 3C). In contrast, the conventional US-guided group had a significantly lower first-attempt success rate (61.1% vs 94.4%; *p* = 0.041). The puncture angle exceeded the 20° threshold in six cases (*p* = 0.019), and vascular injury occurred in five cases (*p* = 0.045; Fig. 3D). There was no significant difference in preparation time between groups (median 15 vs 16 s, *p* = 0.084); however, puncture time was significantly shorter in the D-CEUS–guided group (median 26 vs 45 s, *p* = 0.001).

**Table 1:** Comparison of outcomes between D-CEUS–guided and conventional ultrasound–guided groups

Outcomes	D-CEUS guided (*n* = 18)	Conventional ultrasound guided (*n* = 18)	*p* value a
Preparation time (s), median (minimum–maximum)	15 (12–18)	16 (13–20)	0.084
Puncture time (s), median (minimum–maximum)	26 (18–49)	45 (22–69)	0.001 **
First-attempt success rate, *n* (%)	17 (94.4)	11 (61.1)	0.041 *
Correct puncture rate, *n* (%)	18 (100)	12 (66.7)	0.019 *
Vascular injury rate, *n* (%)	0 (0)	5 (27.8)	0.045 *

D-CEUS = dual contrast-enhanced ultrasound.

a The symbol * indicates statistically significant difference between the two groups.

* *p* < 0.05.

** *p* < 0.01.

**Fig. 5:**
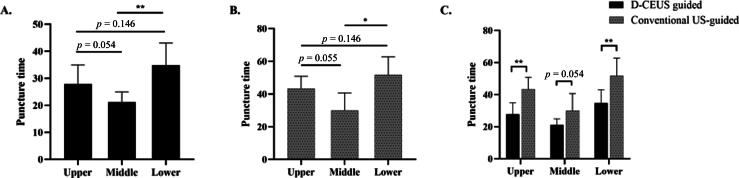
Subgroup analysis of puncture time by calyx location: (A) D-CEUS–guided group, (B) conventional US-guided group, and (C) comparison between groups (D-CEUS vs US). D-CEUS = dual contrast-enhanced ultrasound; US = ultrasound. * *p* < 0.05. ** *p* < 0.01.

Since PCNL puncture complexity depends on calyx location, a subgroup analysis based on puncture time was performed to identify the optimal candidates for D-CEUS. Procedures targeting the upper and, in particular, the lower calyx were more challenging than those involving the middle calyx (Fig. 5A and B). Notably, D-CEUS reduced puncture time significantly for both upper and lower calyceal access, as opposed to conventional US-guided access (Fig. 5C).

## 4 Discussion

This study demonstrates that the novel D-CEUS technique, which combines intravenous and intracavitary contrast injections via the puncture needle, improves the accuracy of percutaneous renal access compared with conventional US. D-CEUS offers two critical advantages: (1) real-time visualization of the puncture trajectory relative to the adjacent vasculature in a nondilated collecting system and (2) precise stone localization through an intracavitary contrast injection via the needle.

Although US-guided PCNL provides real-time imaging and avoids radiation exposure compared with fluoroscopic approaches, its efficacy declines in the absence of hydronephrosis [19]. Reported success rates drop from 98.2% in dilated to 82% in nondilated systems, primarily due to limited anatomical visibility under conventional US [20]. CEUS improves structural visualization in nondilated systems through contrast enhancement. However, existing CEUS-guided techniques rely on retrograde intracavitary contrast, which does not enable the simultaneous visualization of the vasculature and the needle, limiting guidance accuracy during a puncture [11,21,22]. Inadequate calyceal access under these conditions also increases the risk of vascular injury and postoperative hematuria [16,18,23].

The intravascular injection of contrast microbubble agents, which are too large to cross the vascular endothelium, has widely been used in imaging adults across various indications [12,24,25]. IV-CEUS offers real-time, high-resolution imaging with quantitative assessment of both macro- and microvascular perfusion in normal and pathological tissues [12,26]. In this study, IV-CEUS successfully delineated the renal vascular architecture, which was comparable with that seen in preoperative DSA. Leveraging this advantage and using a fully diluted contrast agent, we achieved visualization of the needle and renal cortical vasculature simultaneously. This likely contributed to a higher first-attempt success rate, shorter puncture time, and lower vascular injury rate in the D-CEUS group.

Anatomically, the optimal target for PCNL is the fornix or papilla of the posterior calyx. The access trajectory should be within 20° posterior to the frontal plane of the kidney to minimize bleeding risk and to allow a straight path to the pelvis in the prone position [18]. By assessing puncture accuracy under anatomically optimal conditions, we aimed to explore the full potential of D-CEUS in improving puncture safety and trajectory control. Based on these criteria, the D-CEUS group achieved a 100% correct puncture rate, compared with 66.7% with conventional US. Despite the need for alternative access routes in clinical practice due to variations in stone characteristics and patient anatomy, these results support that D-CEUS may facilitate a safer and more accurate puncture trajectory in PCNL.

Another key advantage of D-CEUS in this study is the direct intracavitary contrast injection through the puncture needle, eliminating the need for a preplaced ureteral catheter. A major limitation of the current CEUS-guided PCNL techniques is the inability to visualize both the renal collecting system and the needle simultaneously in a single image, B-mode visualization is superior to CEUS for visualizing the needle, which complicates puncture guidance [21]. This may hinder precise localization of renal stone. In contrast, D-CEUS enables concurrent visualization of both structures in the CEUS mode, facilitating more accurate needle guidance toward the target stone. Additionally, D-CEUS eliminates the need for ureteral catheterization by visualizing the collecting system via needle injection, thereby improving the overall efficiency of PCNL.

We evaluated the clinical value of D-CEUS with respect to procedure duration, focusing on puncture time. D-CEUS significantly reduced the time from the renal capsule to the target calyx due to enhanced visualization. Although the preoperative steps are more complex and require contrast preparation and venous access, IV-CEUS is a mature, user-friendly technique. In contrast, conventional US often requires additional time to position the needle and identify a safe puncture trajectory that avoids vasculature, particularly in nondilated collecting systems with limited visualization. This likely contributed to the longer puncture time in the conventional group, despite the seemingly more straightforward setup. Notably, a subgroup analysis further confirmed that D-CEUS shortens puncture time significantly for both upper and lower calyceal access. Overall, D-CEUS is a valuable strategy for improving PCNL efficiency, especially for challenging calyceal approaches.

However, this study has several limitations. First, since it is a preclinical animal model, the findings may not be fully translated to humans, and clinical validation is needed. Second, the performance of D-CEUS in obese patients or in the presence of bowel gas—a known limitation of CEUS imaging—was not evaluated [15]. Moreover, while this potential advantage is promising, we acknowledge that the reproducibility of D-CEUS across centers and operators with varying expertise remains to be validated.

To the best of our knowledge, this is the first porcine study to implement the D-CEUS technique with contrast delivered via the puncture needle. These findings suggest that D-CEUS is a feasible technique and a potentially superior alternative to conventional US-guided PCNL, particularly in nondilated systems and anatomically complex scenarios.

## 5 Conclusions

D-CEUS enhances puncture accuracy by allowing real-time visualization of vascular structures and needle trajectory within a nondilated collecting system, increasing first-attempt success and reducing vascular injury. Its unique ability to deliver direct contrast through the puncture needle allows for precise stone localization. These features demonstrate the potential of D-CEUS to improve the safety and efficiency of PCNL.

  ***Author contributions*:** Bingding Huang had full access to all the data in the study and takes responsibility for the integrity of the data and the accuracy of the data analysis.

  *Study concept and design*: Shen, Mai.

*Acquisition of data*: All authors.

*Analysis and interpretation of data*: All authors.

*Drafting of the manuscript*: Shen, Li, Chen.

*Critical revision of the manuscript for important intellectual content*: All authors.

*Statistical analysis*: Liang, C. Zhang.

*Obtaining funding*: X. Zhang, Huang, Mai.

*Administrative, technical, or material support*: Shen, Xie, Mai.

*Supervision*: X. Zhang, Huang, Mai.

*Other*: None.

  ***Financial disclosures:*** Bingding Huang certifies that all conflicts of interest, including specific financial interests and relationships and affiliations relevant to the subject matter or materials discussed in the manuscript (eg, employment/affiliation, grants or funding, consultancies, honoraria, stock ownership or options, expert testimony, royalties, or patents filed, received, or pending), are the following: None.

  ***Funding/Support and role of the sponsor*:** This study was supported by National Natural Science Foundation of China (grant no. 82173259), Beijing Municipal Science and Technology Program for Research and Application of Capital’s Clinical Characteristic Diagnosis and Treatment Technologies (Z221100007422123), Special Project for Disciplinary Innovation and Development of the Chinese People’s Liberation Army General Hospital (2024BJ-13, 2025-CXT-007V), and Shenzhen Science and Technology Program (no. KJZD20240903095605007).
